# Temporal MRI characterization, neurobiochemical and neurobehavioral changes in a mouse repetitive concussive head injury model

**DOI:** 10.1038/srep11178

**Published:** 2015-06-10

**Authors:** Zhihui Yang, Ping Wang, Drake Morgan, Dan Lin, Jianchun Pan, Fan Lin, Kevin H. Strang, Tyler M. Selig, Pablo D. Perez, Marcelo Febo, Binggong Chang, Richard Rubenstein, Kevin K.W. Wang

**Affiliations:** 1 Program for Neurotrauma, Neuroproteomics & Biomarkers Research, Departments of Psychiatry & Neuroscience, McKnight Brain Institute, University of Florida, Gainesville, FL 32611, USA; 2 School of Pharmaceutical Sciences, Wenzhou Medical University, Wenzhou, Zhejiang 325035, P.R. China; 3 Department of Psychiatry, McKnight Brain Institute, University of Florida, Gainesville, FL 32611, USA; 4 Department of Neurology, Box #1213, SUNY Downstate Medical Center, 450 Clarkson Avenue, Brooklyn, NY 11203, USA

## Abstract

Single and repeated sports-related mild traumatic brain injury (mTBI), also referred to as concussion, can result in chronic post-concussive syndrome (PCS), neuropsychological and cognitive deficits, or chronic traumatic encephalopathy (CTE). However PCS is often difficult to diagnose using routine clinical, neuroimaging or laboratory evaluations, while CTE currently only can be definitively diagnosed postmortem. We sought to develop an animal model to simulate human repetitive concussive head injury for systematic study. In this study, mice received single or multiple head impacts by a stereotaxic impact device with a custom-made rubber tip-fitted impactor. Dynamic changes in MRI, neurobiochemical markers (Tau hyperphosphorylation and glia activation in brain tissues) and neurobehavioral functions such as anxiety, depression, motor function and cognitive function at various acute/subacute (1-7 day post-injury) and chronic (14-60 days post-injury) time points were examined. To explore the potential biomarkers of rCHI, serum levels of total Tau (T-Tau) and phosphorylated Tau (P-Tau) were also monitored at various time points. Our results show temporal dynamics of MRI consistent with structural perturbation in the acute phase and neurobiochemical changes (P-Tau and GFAP induction) in the subacute and chronic phase as well as development of chronic neurobehavioral changes, which resemble those observed in mTBI patients.

Sports-related mTBI (also referred to as concussion) has recently attracted increasing public health awareness. The Center for Disease Control estimates that between 1.6 and 3.8 million recognized concussions are sustained from sport and recreational activities every year in the United States alone^1^,^2^. To date, full clinical pictures of such patients have not yet been established. In general terms, sports-related mTBI has post-concussive syndrome (PCS) that include headache, dizziness, anxiety, drowsiness, loss of concentration and memory while traditional magnetic resonance imaging (MRI) and computerized tomography (CT) scans are often normal. Although most patients with concussion recover well, growing evidence indicates that multiple concussions are less benign. In fact, repetitive mild closed head injury (rmCHI) have been linked to the development of chronic traumatic encephalopathy(CTE), a progressive degenerative disease^3^. Gross pathological changes of rmTBI have also been reported, including long-term persistent brain volume loss, as well as histological changes, including Tau-immunoreactive neurofibrillary tangles, the hallmark of CTE, in the brain found in athletes (and others) with a history of repetitive brain trauma^4^. These changes in the brain can begin months, years, or even decades after the last concussion or end of active athletic involvement. Unlike severe TBI victims that are relatively easy to diagnose since they suffer clear pathologies such as direct brain tissue damage, rupture of the barrier (BBB), and post-injury edema, patients sustaining chronic mTBI or PCS are difficult to diagnose definitively with routine neuroimaging tests and laboratory evaluations. Currently, CTE can only be definitively diagnosed through post-mortem examination of the brain (such as the wide-spread presence of Tau-aggregates), although efforts are underway to learn how to diagnose CTE pre-mortem^5^.

In essence, concussion is thought to be a temporary disruption of brain function that typically resolves spontaneously; however, some patients can have persistent neurological sequelae. The spectrum of sequelae after rmTBI is important to consider when translating clinical experience to the bench. There has been a recent focus on developing animal models of mTBI including mild closed head injury (mCHI). In CHI models, the injury is induced through the intact skull by direct impact (e.g. dropping a weight on the intact skull or striking the intact skull with a piston), non-impact (e.g. blast) or inertial loading (by rapid rotation of head in the sagittal, coronal or oblique planes)^6^. Although these models have demonstrated some degree of behavioral deficits and some histological changes, no systematic study was reported to establish the dynamic correlation between brain morphology or biochemical changes and the development of functional deficits. In addition, even though Tau pathology is considered as a hallmark of CTE progression, there are no pre-clinical reports monitoring changes of this potential biomarker from the acute to chronic stages.

We sought to address this through the use of a single (s) and repetitive (r) mCHI mouse model with the purpose of making it as clinically relevant as possible, in which no remarkable tissue disorganization, or subdural or interstitial hemorrhage in cortex, hippocampus, cerebellum and brain stem was found by histological examination. Then dynamic changes in MRI features, neurochemical (Tau phosphorylation and glia activation) as well as neurobehavioral changes such as anxiety, depression, motor function and cognitive function were monitored at various acute (1-7 day after last injury) and chronic (30 and 60 days after last injury) time points. Furthermore, the levels of T-Tau and P-Tau in serum were dynamically detected using a novel ultrasensitive assay as we previous reported^7^.

## Results

To setup an rmCHI model which is as clinically relevant as possible, no skull fractures and subarachnoid, epidural or subdural hemorrhage were expected. The brain sagittal sections from sham, mCHI, and rmCHI mice were histologically normal and showed no remarkable tissue disorganization, or subdural or interstitial hemorrhage in cortex, hippocampus, cerebellum and brain stem by hematoxylin and eosin staining (H&E staining, data not shown). This repetitive concussion model has low risk in producing hypoxia or hypotension, edema, significant BBB damage, or acute and chronic cell death/tissue loss.

### Diffusion weighted Imaging and T2 relaxometry shows delayed cortical and hippocampal alterations

**Figure 1:**
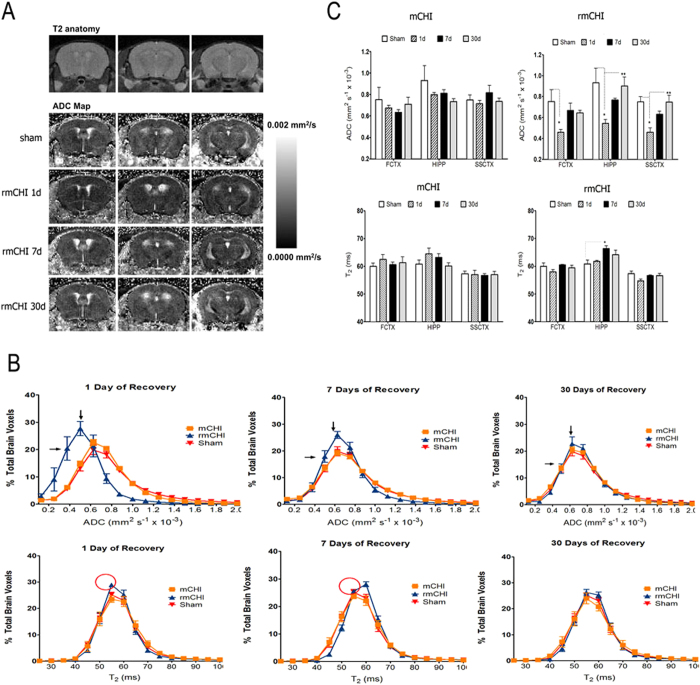
Altered brain morphology in repetitive closed-head injury (rCHI). (**A**) Representative ADC maps of repeated closed head injury (rCHI) at 1, 7 and 30 days of recovery. Top row shows a representative T2 weighted anatomical scan. From top to bottom are the sham condition and 1, 7 and 30 days post injury. Scale bar on the right indicates image threshold for voxel diffusion values are between 0 and 0.002 mm2/sec. **(B)** Histograms showing the distribution of whole brain voxel ADC (upper) and T2 (lower) values for sham mice and mice sustaining rCHI. Mice were imaged following 1, 7 and 30 days of recovery from initial trauma to the head. Data are expressed as % total brain voxels (± SEM) for several ADC or T2 values. Arrows (upper panel) highlight histogram shifting in rCHI animals from low ADC on day 1 to average values on day 30. Circles (lower panel) also highlight differences between rCHI and other conditions. **(C)** ADC and T2 (upper and lower respectively) values for frontal cortex (FCTX), hippocampus (HIPP) and sensorimotor cortex (SCCTX) for various TBI conditions. Data are expressed as mean ADC or T2 values (± SEM). *Significantly different from sham condition; **significantly different from 1 day recovery condition (MANOVA with Sidak’s multiple comparison post-hoc test, p <0.05).

We first analyzed whole brain of apparent diffusion coefficient (ADC) and T2 relaxation values by plotting histograms across the single/repetitive concussion and comparing these to sham controls (Fig. 1). The results indicate that rmCHI showed an initial lowering of ADC values (0.4–0.6 × 10^−3^ mm^2^/s) below that shown by the other two conditions (mCHI and sham). This was observed following only 1 day after last injury. At 7 days post-mCHI there was still a difference between these conditions, albeit much less in magnitude (Fig. 1B, top). The difference between the various conditions is completely gone by 30 days post last injury. A similar pattern of TBI-induced change was not apparent for whole brain T2 relaxation values (Fig. 1B, buttom). We did find a slight but significant decrease in T2 relaxation at day 1 after last injury in rmCHI mice, which shifted to a slight increase by day 7. No difference was observed by 30 days of recovery. Current consensus statements suggest that the prototypical recovery pattern for an uncomplicated sports concussion may show nearly complete improvement in the first 1–2 weeks post-mTBI, although some symptoms may persist for several weeks^8^. Clinical MRI is recommended only if a more serious brain injury is suspected, or if symptoms do not clear within the first 7 to 10 days. Our data suggest clinical correlates for mCHI in this model in which brain morphology alterations were spontaneously improved after 7 days of recovery.

In order to examine the regional differences between conditions, especially rmCHI which showed a significant effect on day 1 of recovery, we analyzed ADC and T2 values slice-by-slice (MRI slice coverage included the most rostral extent of the prefrontal cortex to the more caudal cerebellar/brainstem areas). This intermediate analysis showed that the likely range of slices affected by repetitive concussive head injury was between slices 6-8 which included the sensorimotor cortical (SSCTX) area directly impacted by this type of lesion and the hippocampus (HIPP). ROI analysis was then carried out which included these regions and the frontal cortex (FCTX). We observed that in all of these regions there is an initial reduction in ADC at 1 day after rmCHI. The lower ADC recovered partially by day 7 and recovered fully by day 30 (Fig. 1C, top). This pattern of altered ADC values was not observed for single CHI conditions. Interestingly we observed that while T2 values per ROI was not altered in the same fashion, there was also an increase in T2 at day 7 post-injury only for the rCHI hippocampus. Therefore, this might indicate a subacute neurobiochemical event occurring at this time point that could involve an increase in T2 relaxation values (Fig. 1C, bottom).

### rmCHI induced short-term impairments in motor and neuromuscular function.

**Figure 2:**
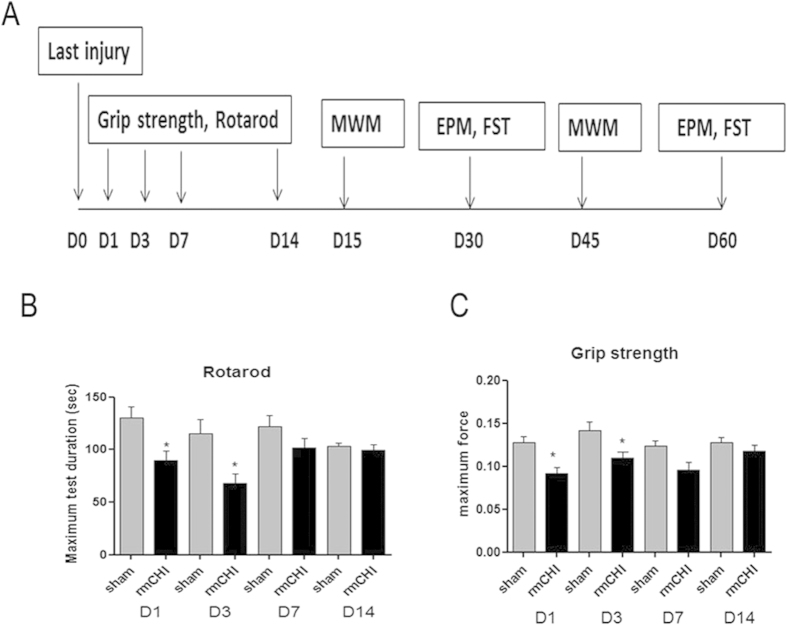
rmCHI-induced short-term impairments in motor and neuromuscular function. (**A**) Schematic showing schedule of behavioral tests employed in this study at the following time points after injury: grip strength and rotarod, 1, 3, 7 and 14 days post injury; Morris Water Maze (MWM), 15 and 45 days post injury; elevated plus maze (EPM) and forced swimming test (FST), 30 and 60 days post injury. We used rotarod testing to evaluate motor coordination (**B**) and grip strength testing to measure neuromuscular function (**C**). Data are means ± SEM; n = 12 per group. *P < 0.05 compared with sham (two tailed t student test)

Since in professional or recreational impact sports, multiple concussions or multiple “sub-concussions” are very common, we focused on the functional outcome for rmCHI mice. We used rotarod test to evaluate motor coordination (Fig. 2). The results showed significant (P < 0.05) deficits in motor performance (time spent on the rotarod in seconds) in mice after rmCHI (89.41 ± 9.43, 68.2 ± 8.99 at D1 and D3 post-occlusion, respectively) compared to sham controls (130.28 ± 10.58, 114.88 ± 14.20 at D1 and D3 post-occlusion, respectively) (Fig. 2B). By 7 days post-injury, the mice regain the ability to remain on the rotarod and there was no difference in time spent on the rotarod at 14 days post-injury compared to sham controls. Possible impairment neuromuscular performance was also assessed with a grip strength test (Fig. 2C). Grip-strength scores showed a similar pattern of rmCHI-induced deficit in the neuromuscular function to motor coordination. Grip-strength decreased significantly in mice subjected to rmCHI at 24 and 72 hours post-injury, and the neuromuscular function improved after 7-day recovery. The mice also completely recovered their grip strength by 14 days post- injury.

### rmCHI affects neuropsychological behaviors

To determine potential neuropsychological behavioral changes after rmCHI, an elevated plus maze (EPM) test was performed to investigate the anxiety-like behavior while the forced swim test (FST) was used to determine depression-like behavior.

**Figure 3:**
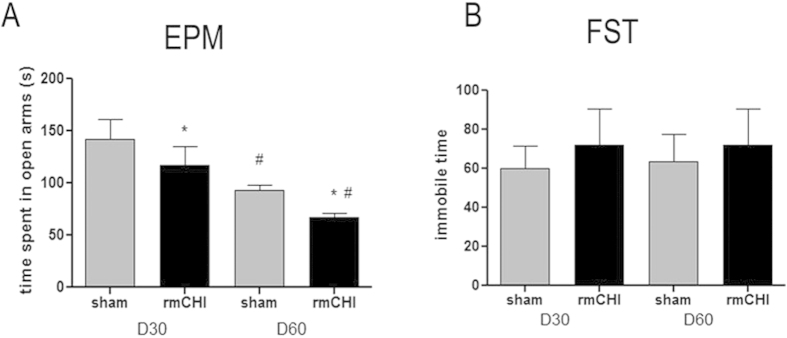
rmCHI affects neurobehavioral functions. Anxiety-related behavior was measured by elevated plus maze test (EPM) **(A)** and depression-related behavior was monitored by forced swimming test (FST) **(B)** at 30 and 60 days after injury. Data represents mean ± SEM; n = 12 per group, ^*^p < 0.05 shows significant difference for rmCHI group as compared with corresponding sham (t test). ^#^p < 0.05 shows significant difference for day 30 group as compared with corresponding Day 60 group (t test).

Specifically, repetitive concussions (rmCHI) resulted in significantly reduced time spent in the open arms of the maze in the EPM test at both Day 30 and Day 60 post-injury when compared to respective sham control groups, consistent with increased anxiety-like behavior (Fig. 3A, indicated by *). Interestingly, both sham and rmCHI groups actually spent less time in open arm at Day 60 than at Day 30, suggesting a neuroadaptive behavior (Fig. 3A, indicated by #).

In FST, although there was an apparent trend of increased immobility time in rmCHI mice compared to control mice at both 30 and 60 days post-injury, no statistical significance was found (Fig. 3B). Based on our results, anxiety-related behavior but not depression-related behavior was found in rmCHI mice at 30 and 60 days post-injury.

### Time-dependent spatial learning and memory deficits in brain-injured mice

**Figure 4:**
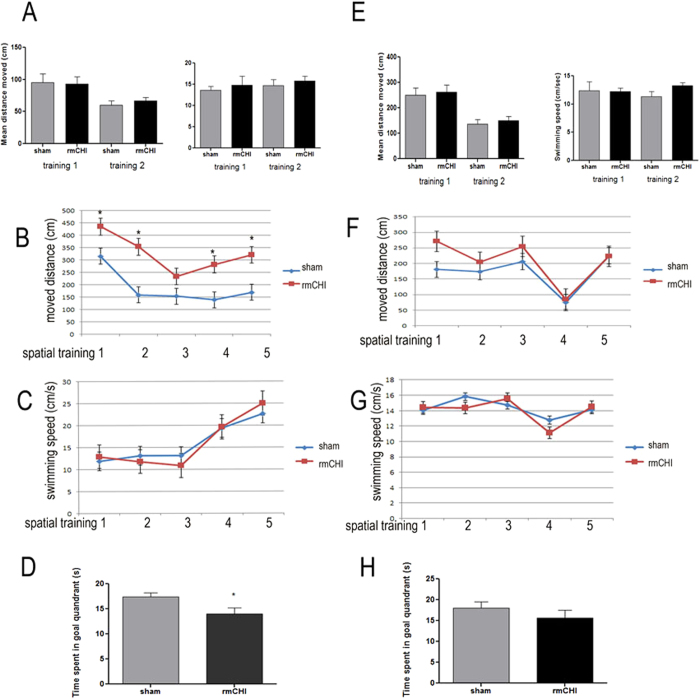
Spatial learning and memory deficits in the Morris water maze test in mice after rCHI. Morris water maze test were performed at 15 days (**A-D**) and 45 days post injury (**E-H**). Panel (**A**) and (**E**): bar graph show path length (cm) and swimming speed (cm/s) over the 2 days of cue training. (**B**) and (**F**): Spatial learning curve for MWM acquisition trials across a period of 5d (hidden platform moved to a new location). (**C**) and (**G**) show swimming speed (cm/s) over the 5 days of acquisition trials. (**D**) and (**H**) show time (s) spent in the target quadrant during the interpolated 30 s probe trials in which the platform was removed from the tank (probe trails). Data are presented as mean ± SEM. * represents P < 0.05.

To determine whether the rmCHI led to cognitive and memory impairments in our mouse model, we assessed hippocampal-dependent learning and memory using the Morris Water Maze (MWM) test. Figure 4 showed motor function deficit in rmCHI mice disappeared after 14 days of recovery. To avoid false-positive results by motor ability, MWM started from day 15 after last injury and repeated one month later to test longer lasting effects of rmCHI. In this task, animals were trained to find the hidden platform in a pool of opaque water. Prior to the training, two-day cue training was used to assess visual acuity and motor ability of the mice to escape to the platform independent of their spatial learning ability. Mice were then trained and learned the location of the platform by using visual cues outside the maze for 6 days. During the cue training the control and injured mice showed no difference in distance traveled to reach the visible platform or overall swimming speed (Fig. 4A,E), suggesting that there are no significant motor or visual impairments that would explain any learning and memory deficits with brain injury. During the acquisition phase of the MWM task, even though both sham and day 15 post-rmCHI groups showed daily improvements in their ability to locate the hidden platform, rmCHI mice demonstrated longer path lengths to the platform than sham mice suggesting that these mice exhibited significantly impaired spatial learning capacity (p < 0.05, two-way ANOVA) (Fig. 4B). At 45 days post-injury, the time to reach the platform by both the rmCHI group and sham group are not significantly different (Fig. 4F). For the probe trial testing, rmCHI animals at day 15 exhibited impaired memory deficits, committing significantly less time in the target quadrant compared to sham mice (Fig. 4D). By 45 days, the rmCHI mice also showed a trend towards reduced time spent in target quadrant (Fig. 4H).

### Neurobiochemical changes after rmCHI

**Figure 5:**
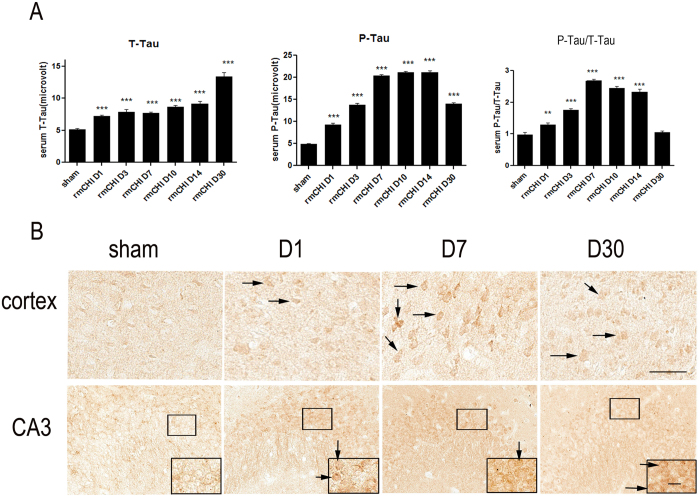
Tau and phosho-Tau changes after repetitive closed head injury. (**A**) Detection of Tau by a-EIMAF in mouse serum following rCHI. Mice were subjected to repetitive mTBI and blood was collected at various time points as indicated. The levels of T-Tau (left) and P-Tau (middle) as well as the ratio of P-Tau to T-Tau (right) in serum were determined by a-EIMAF. Statistical analysis (t-test) was based on comparison to naïve: ** p < 0.01; *** p < 0.0001. (**B**) Immunostaining of mice brain sections using P-Ser202 (CP13) antibody to assess changes in tau. Representative images obtained from mouse brain sections showing P-Tau immunoreactive cells in the cortex and hippocampal CA3 pyramidal neurons at different time points post-rCHI. The insert show magnification of the dense P-Tau stained cells in each section. Scale bar = 50 μm. Arrows indicated representative P-Tau positive cells in each section.

Tau protein is a microtubule-associated protein exclusively located in axons. But Tau protein (total Tau, T-Tau) release to biofluid could be considered an index of diffused axonal injury. In addition, phosphorylated tau (P-Tau) is a pathological marker for CTE. T-Tau and P-Tau levels in mouse serum were measured post-mTBI using the ultra-sensitive immunoassay platform—Enhanced Immunoassay using Multi-Arrayed Fiberoptics (EIMAF) in combination with rolling circle amplification (a-EIMAF). Mice were subjected to rmCHI and followed by a-EIMAF assay over a 30-day period during which changes in serum levels of T-Tau and P-Tau were determined. At each time point, serum was collected from separate groups each consisting of 5-8 mice and serum samples were analyzed individually in triplicate, in a blinded fashion. We observed that there was a baseline level of T-Tau in control mouse serum. However, the levels of serum T-Tau began to increase gradually after injury and it remained significantly higher than sham controls from day 1 to day 30 (Fig. 5A). Compared to T-Tau, the increases in P-Tau were even more dramatic over the 14 day post-injury period followed by a decline. The increases of P-Tau levels over sham controls were significant. When analyzing the P-Tau/T-Tau ratio, we also found that the ratio increased significantly and reached a peak at 7 days after which it began to decrease and returned to normal at the 30-day time point (Fig. 5A).

To explore the involvement of Tau phosphorylation we then examined the expression profile of P-Tau at the p-Ser202 site in mouse brain sections. As shown in Fig. 5B, few of P-Tau (Ser-202) immuno-positive cells were observed in cortex, and hippocampal CA3 region from sham mice. P-Tau immuno-positive cells in cortex of rmCHI mice became more pronounced than sham mice at 24 hr, as well as 7 and 30 days after injury (Fig. 5B). We also observed an increase of P-Tau positive hippocampal CA3 pyramidal neurons (probed with anti-P-Tau Ser202 antibody) at days 1 and 7 post-rmCHI. However by 30 days post-injury, much fewer P-Tau positive pyramidal neurons were observed. The overall immunohistochemistry changes of P-Tau are consistent with the pattern of P-Tau changes observed in serum (Fig. 5A).

### Biochemical marker of gliosis was increased after rmCHI

**Figure 6:**
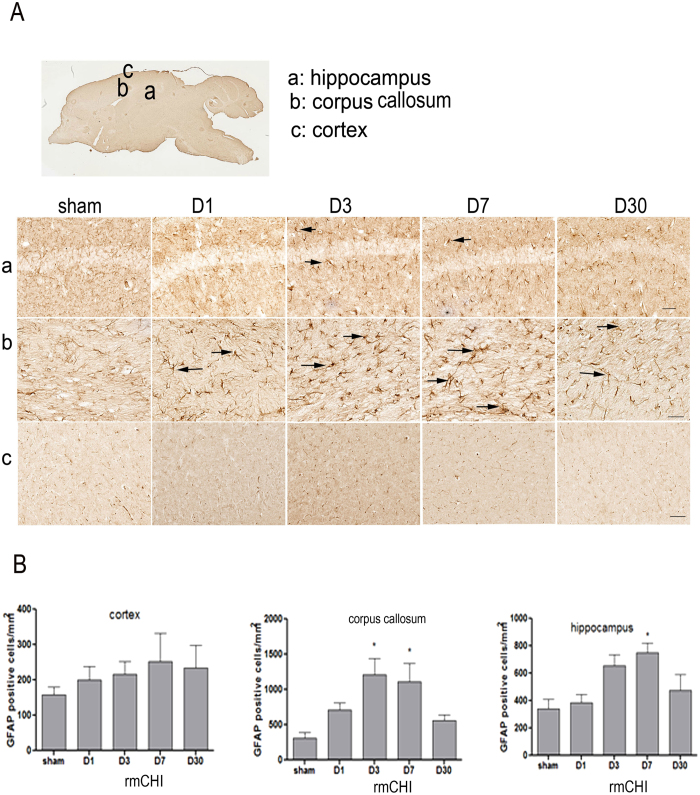
Increase of biochemical marker for gliosis (GFAP) after repetitive closed head injury. (**A**) Schematic diagrams of brain sections. (**B**) Representative images were taken from cortex (upper), corpus callosum (middle) and hippocampus (lower). **(C**) GFAP immunoreactivity was quantified as the number of immunoreactive cells per tissue area (mm^2^). Arrows indicated representative astrogliosis. N = 6 mice/group. Data are presented as mean ± SEM. * represents P < 0.05 by t-test.

Astrocytic activation and proliferation is thought to contribute to the pathogenesis of TBI. We next assessed posttraumatic astrocyte activation status in the brain of sham and rmCHI mice. In response to repetitive injury, cellular processes of activated astrocytes show signs of hypertrophic appearances with thick, densely labeled processes, and large cell bodies. In mice exposed to rmCHI, there was prominent astrogliosis throughout the corpus callosum and hippocampus at days 3 and 7 post-injury (Fig. 6A). We performed stereologic assessment of GFAP-immunoreactive cell number in the injured cortex, corpus callosum and hippocampus of each group. Semi-quantitative estimation revealed that the GFAP immunoreactivity was significantly increased in these regions from 3 days post-injury and lasted at least 7 days compared with the sham group (Fig. 6B–D). Interestingly, there was significant astrogliosis in injured cortex after mild direct force to the head, although there was a trend towards an increase over time – this is consistent with findings from other researchers^9^.

## Discussion

**Figure 7:**
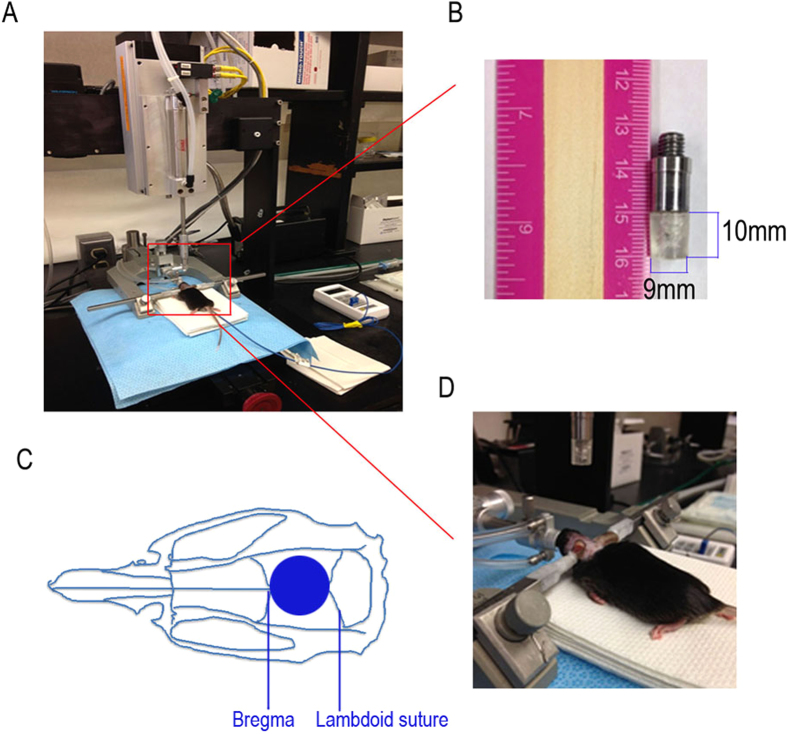
A mouse model of mild closed-head injury (CHI). (**A**) a mouse in the stereotaxic frame with the impactor aligned above the head. (**B**) Custom-made rubber tip measuring 1 cm in thickness and 9 mm in diameter. (**C**) The center of impact (see circle) corresponds to the central sagittal suture midway between coronal and lambdoid sutures. (**D**) The injury phase: impact depth: 3.8 mm; dwell time: 200 ms; impact speed: 4 m/s. For repetitive injuries, identical impact procedures were performed for 4 times at an interval of 48 hours. For sham injuries, the same procedure was performed except the impact.

In this study, we have improved and characterized a mouse model of concussive rmTBI using a novel stereotaxic impact device. Improvements of the stand model came from the development of a custom-made 10 mm thick silicon rubber tip (instead of widely used metal tips or rubber coated-metal tip), which minimized the possibility of inducing skull fracture and the injury degree. (Figure 7). This new method better models mild concussive brain injury that affects millions. In human, signs of a concussion may include brief loss of consciousness (LOC) after the injury, memory problems, confusion, drowsiness or feeling sluggish, dizziness, double vision or blurred vision, headache and nausea or vomiting. LOC is relatively rare and occurs in less than 10% of concussions. In sports-related concussions, prolonged LOC lasting longer than 1 to 2 minutes is much less frequent, with most LOC lasting less than a minute in duration^10^. In our model, although some mice were found LOC by brief loss of breathing (less than 15 s), no mice was observed to show gross clinical complications, such as tissue damage to chromodacryorrhea and severe evidence of subdural hemorrhage and intracerebral bleeding, which in itself could only be categorized as moderate to severe closed-head injury.

Our findings also show promising result that early diagnostic imaging applications like diffusion weighted imaging and T2 relaxometry can help identify neurostructural perturbations early after rmCHI (Fig. 1). In this study we observed reduced ADC values at 24 hrs in the sensorimotor cortex, frontal cortex and hippocampus regions in the concussed mice when compared with the sham control mice (Fig. 1). Reductions in tissue diffusion values can reflect underlying microstructural changes involving greater restrictions to bulk water displacements within the brain tissue. This is opposite to what would be observed with an increased ADC and the occurrence of edema or swelling, which is unlikely to have occurred here with rmCHI. Whether or not this occurs in white versus grey matter was not studied here but will be the focus on future studies. Sensorimotor cortex is an area of the cerebral cortex related to somatosensory and motor functions. Sensorimotor cortex has been implicated in determining the organization and representation of conceptual knowledge of concrete objects and actions^11^. Altered sensorimotor cortex may be associated with the motor deficits shown as reduced time on an accelerating rotarod (Fig. 2). The frontal cortex contains most of the dopamine-sensitive neurons in the cerebral cortex. The dopamine system is associated with reward, attention, short-term memory tasks, planning, and motivation. Frontal cortex injury occurring in TBI patients was reported to be diagnosed as Alzheimer’s disease or Parkinson’s disease following the accident. The hippocampus is well-known to be associated primarily with memory, in particular long-term memory. The frontal cortex and hippocampus alteration may contribute to the cognitive deficits observed in the present study (Fig. 4). Our results using MRI indicated that the brain morphology alterations were spontaneously improved at day 7 and disappeared after a long-term recovery (30 days). The dynamic pattern of MRI measurements is consistent with that of the motor and neuromuscular function in that these deficits were improved after 7 days of recovery and disappeared at day 30. The altered frontal cortex and hippocampus appear to be improved while the behaviors tests till show learning and memory deficits. Although how those alterations shown in MRI reflect cognitive deficits are not fully understood yet, the possibility of microstructural changes or neurodegeneration cannot be ruled out. Thus, the region-specific structural changes observed with dynamic MRI measurements are consistent with the observed behavioral deficits, and recovery patterns with rmCHI. It will be of importance in future studies to examine functional changes in neural activation as consequence of the structural changes.

PCS including emotional liability or irritability, cognitive decline and/or depression and anxiety may persist beyond the expected recovery period. We therefore sought to examine some of these post-concussive parameters in our mouse rmCHI model. With the MWM test, we demonstrated deficits in both spatial learning capacity and memory at day 15. However, such deficits were improved at day 45 (Fig. 4). Even though other researchers observed long-term cognitive deficits, for example, 6 months after repetitive injury^9^, it is important to note that severity of injury (mild, moderate vs. severe), times of injury, interval between multiple injuries are major factors that may have contributed to the inconsistency between findings. Anxiety-related symptomatology is also one of the psychological factors that often observed in individuals following a sports-related concussion or mTBI. In the present study, we observed long-lasting anxiety-like behavior (at least 60 days post injury) although depression-like behavior in repetitive mTBI mice did not reach significance (Fig. 3). One plausible interpretation of these findings is that the structural MRI changes directly related to the brain injury may, in turn, place individuals at greater risk for resultant anxiety symptoms, especially after sustaining repetitive injuries^12^,^13^. Interestingly, anxiety-like behavior is also a symptom in CTE patients. TBI often involves damage to the prefrontal cortex, ventral frontal lobe and anterior temporal lobe, areas which are heavily implicated in the recognition of emotionally-relevant stimuli and regulation of the reactions to those stimuli^14^,^15^. Some patients who have experienced frontal lobe trauma may also exhibit depression, anxiety or other psychological deficits^16^. In preclinical research, hippocampal volume loss was found significantly associated with anxiety^17^ . However whether early perturbation of frontal cortex and hippocampus will contribute to the later behavioral disorders we observed need further studies.

The neuro-inflammatory responses generated by the activated astrocytes can disturb synaptic plasticity and may cause a decline in cognitive function^18^,^19^,^20^. Our data showed initial astrogliosis at day 1 post-rmCHI and peaking at days 3 and 7 throughout the corpus callosum (Fig. 6). Although there was no statistical difference at day 30, activated glia remained observable (Fig. 6). In hippocampus, a similar pattern was found in astrogliosis suggesting astrocytic activation contributes to cognitive impairment. Additionally, increased P-Tau in injured brain might be another factor associated with the cognitive deficits observed (Fig. 5). rmCHI resulted in a marked increase in P-Tau notably in the superficial layers of the cortex and the hippocampal CA3 region. Multiple concussions can predispose individuals to develop a pathology distinct from of tauopathy-related early onset dementia. Additionally, Tau pathology after TBI creates a possible molecular link between TBI and development of CTE. Our data suggest this model may be suitable for biomarkers exploration as well as evaluating potential therapeutic interventions for mTBI. Increased Tau levels have been reported in the CSF and serum following TBI^1^,^2^,^9^ and also show promise as a specific serum biomarker in both human patients and in experimental models. However, most assays were performed on severe TBI subjects or animal models^21^,^22^. In addition, the temporal progression of changes in serum Tau levels has not been extensively evaluated. In our previous studies, we report the dynamic pattern of Tau levels in serum from humans with severe TBI and in severe injury TBI animal model (controlled cortical impact)^21^. Here we report for the first time the pattern in a mTBI mouse model. We found that serum P-Tau levels gradually increased from days 1 to 7 and maintained the high levels during days 7 to 14. After that there was a dramatically decrease in the P-Tau levels. In contrast to P-Tau, T-Tau generally increased to day 30 (Fig. 5). The increased T-Tau pattern is similar to our previous studies with controlled cortical impact rodent models. However, compared to a sustained increased P-Tau levels in severe injury models, mild injury shows a significant decrease during the chronic stages (e.g. day 30). Due to higher levels of T-Tau which are maintained at later time points during the chronic stage, whether the reduced P-Tau levels at the later time points during the later stages is related to a lower risk of developing a neurodegenerative disease is unknown at this time. Further studies need to address this question. In addition, we performed GFAP and p-Tau immunohistochemistry in single mTBI mice, and no significant changes were found between sham controls and mTBI mice at various time points (data not shown). These findings are consistent with our MRI results that there was no significant alteration observed following a single injury.

In conclusion, our results show temporal dynamics of MRI characterization and neurobiochemical changes are generally consistent with development of neurobehavioral disorders and possible tauopathy-linked neurodegeneration. Repetitive mTBI induces short-term brain structural and histological alterations, motor impairment as well as learning and memory impairments, and profound and anxiety-like behaviors, which resemble those observed in human mTBI patients. One limitation of this study is that, we have not fully investigated the possibility of subtle neuronal degeneration in this model that may be related to the behavioral disorders. Additionally, although Tau pathology is considered as a hallmark of CTE, the long term time-course of T-Tau and P-Tau levels in serum and in tissue as the mice aged after rmCHI have not been established. In addition, based on this model, future investigations into the dynamics of other candidate TBI biomarkers including GFAP, S100β, TDP-43 and UCH-1^23^,^24^,^25^,^26^,^27^, which are detectable in serum and/or CSF in humans with severe TBI, will also be of great interest.

## Materials and Methods

### Animals

The experiments were approved by the Institutional Animal Care and Use Committee of University of Florida, and were in accordance with the National Institutes of Health Guide for the Care and Use of Laboratory Animals. Male C57BL/6J mice (Charles River Laboratories, Raleigh, NC, USA) were used. Bedding, nesting material, food and water were provided *ad libitum*. Ambient temperature was controlled at 20-22 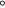
C with constant 12-hr light/12-hr dark cycles. Mice were 3-4 months of age at the beginning of experiments. All behavioral testing was performed in isolated behavior rooms. In MRI experiments, 6 mice per group were included. In all behavioral tests including accelerating rotarod, grip strength test, FST, EPM and MWM) 12 mice per group were used. In immunohistochemistry, 5 animals per group were included. In serum T-Tau/P-Tau analysis, samples from 6-8 mice per group were included.

### Induction of mCHI

The mice were selected at random to be exposed to single mCHI or rmCHI (one or four impacts with a 72 hr interval between impacts) or no injury. On day 1, mice were anesthetized with 4% isoflurane in oxygen as a carrier gas for 4 min followed by maintenance anesthesia of 2% to 3% isoflurane. After reaching a deep level of anesthesia, the mice were shaved and mounted in a stereotactic frame in a prone position. Brain trauma was produced using a PSI TBI-0310 Impactor (Precision systems and instrumentation, LLC, USA) by impacting the sagittal suture midway with a commercial rubber impactor tip (1 cm in thickness, 9 mm in diameter) at a velocity of 4.0 m/s, 3.8 mm compression depth and a 200 ms dwell time (compression duration). The center of impact corresponds to the central sagittal suture midway between coronal and lambdoid sutures. The deformation of the rubber tip spread the impact force over the skull. For the rmCHI mice, additional injuries were administered at days 4, 7 and 10after the initial injury. Sham control animals underwent identical procedures, but did not receive an impact injury (Fig. 7).

### MRI and Image Analysis

During imaging sessions, mice were anesthetized under 1.5% isoflurane gas, placed in a body tube cradle and setup in a surface transmit/receive radio frequency coil system used for high-resolution imaging on a Magnex Scientific 4.7 Tesla MR scanner. Diffusion weighted imaging (DWI) and T2 relaxometry pulse sequences were run on a VnmrJ 3.1 console (Agilent, Palo Atlo, CA). Respiratory rates and core body temperature were monitored continuously throughout the experiments. Scanning lasted 1 hr, with 30 min for diffusion and 30 min for T2 scanning per mouse. For diffusion imaging, we collected one B0 image with an additional 3 diffusion-sensitized images along the read, slice and phase encode directions using a conventional spin echo sequence. The following parameters were used for DWI: echo time (TE) = 31 ms, repetition time (TR) = 2000 ms, maximum b value = 900 s/mm^2^, field of view 24 mm^2^ along the read, phase directions and 1 mm along the slice direction, and data matrix of 128 × 128 × 10 slices. For T2 relaxation the same image dimensions applied for DWI were used, the TR was 3000 ms and 20 TE’s were used for reconstruction of T2 maps (TE’s in ms: 10–200 at 10 ms intervals. Signal averaging was used to increase signal to noise for both diffusion and T2 scanning.

Images were imported into NIH Image J (rsbweb.nih.gov/ij) for processing of apparent diffusion coefficient (ADC) and T2 maps. For ADC maps the B0 image and a single diffusion direction image was used (phase encode) for reconstruction of ADC maps. This direction had minimal artifacts compared to the other diffusion directions. T2 maps were reconstructed from a log linear regression of the multi-TE value datasets. A three-step approach was taken for analyzing image data obtained from manually drawn regions of interest (ROI). ROI’s were manually delineated using ITK-Snap program. First a whole brain ROI was traced for each subject and voxel signal intensity histograms were graphed in order to determine gross changes in both ADC and T2 across conditions. The next step involved a slice-by-slice ROI procedure that was used to determine the coronal brain slice range consistently showing any injury associated abnormalities in ADC and T2 compared to controls. Finally, 4 regions of interest were drawn based on the first 2 steps, which included regions that could potentially be affected by the type of TBI procedure employed. The ROI’s included the frontal cortex, brainstem, the hippocampus and the sensorimotor cortical region corresponding to the site of closed head compression. ADC and T2 values were exported for statistical analysis in Graphpad Prism 6.0. The range of measured ADC and T2 values and quantitative maps were consistent with those reported for mouse brain in the literature (Fig. 1). The groups were control animals (sham), mCHI or rmCHI with 1, 7, and 30 day recoveries, (each condition and day had 5-7 adult male C57BL/6J mice). A multi-way analysis of variance (ANOVA) with Sidak’s multiple comparisons post-hoc test was used (*p* = 0.05).

### Accelerating Rotarod

To measure motor function, an accelerating rotarod (model ROTAMEX-5, Columbus Instruments, Columbus, OH) was used for quantitative analysis of motor coordination and learning. The rotarod consisted of a plastic rod (diameter = 3 cm; length = 30 cm) partitioned off with round plates to prevent the mice from escaping from the sides of the rod. The rod was covered with smooth plastic tubing and suspended 16 cm above five plastic levers attached to timers that immediately stop when mice fall from the rod. The mice were placed on the rod that rotated at 2 rpm at 0 min and accelerated to 40 rpm after 5 min of run time. Mice were oriented perpendicular to the long axis of the rod, such that the mice had to make forward walking movements to avoid falling. At 24 hr, 3 and 7 days post last injury, each mouse received three training trials with a 10 min inter-trial interval followed by three “test” trials on which latency time to fall from the rod was measured and then the average latency time was used.

### Grip Strength Test

Grip strength tests were performed using a grip strength meter from Columbus Instruments (DFIS-2 Series Digital Force Gauge, Columbus Instruments, OH). Grip strength testing was performed by allowing the animals to grasp a thin bar attached to the force gauge. This was followed by pulling the animal away from the gauge until the forelimbs released the bar. This provides a value for the force of maximal grip strength. The force measurements were recorded in five separate trials and the maximal grip strength was used in analyses.

### Morris water maze (MWM)

MWM consists of a 4-ft diameter pool filled with water (24–26 ^o^C) clouded by non-toxic paint. The pool was divided into four quadrants, each with a platform position equidistant from the center to the wall. During cue training that was used to assess visual acuity and motor ability of the mice to escape to the platform independent of their spatial learning ability, the pool was filled to 1 cm below a visible plastic platform. During the spatial reference memory assessment (hidden platform training), the platform (12 cm diameter) was located in the southwest quadrant of the maze and submerged 1 cm below the surface of the water. During cue training, the platform and start positions were varied on each trial. The protocol was used as previous reported^28^. Mice were given 6 trials with interval of 10 min for two consecutive days. Beginning on the day after cue training was completed, mice received 5 consecutive days of hidden platform training (4 trials/day) to a hidden platform to assess spatial reference memory. The animals were allowed to search for the hidden platform for a period of 60 sec, and distance length to reach the platform was recorded. If an animal failed to find the hidden platform on any given trial, it was led there by the experimenter. As in cue training, mice were given a 10 min inter-trial rest interval between trials for both training and probe trials. The start position for each trial (north, south, east, and west) varied on each trial. In the last day animals were tested in a probe trial in which the platform was removed from the pool and allowed to search for a period of 30 sec. Swimming time in target quadrant, where the platform was placed was recorded. Each mouse’s swimming episode was tracked and analyzed using a computer-based video tracking system (EthoVision XT 7.0, Noldus Information Technology Inc, Leesburg, VA, USA).

### Elevated Plus Maze

The elevated plus maze consisted of two open arms and two closed arms. Rodents avoid the open arms of the plus maze so that decreased time spent in and decreased entries into the open arms are thought to reflect an enhanced level of anxiety. Mice were placed individually in the center of the maze (each arm was 33 cm long and 5 cm wide with 25 cm high walls on closed arms) and allowed free access for 5 min. Animals spent time either in a closed, safe, area (closed arms), in an open area (open arms) or in the middle, intermediate zone. Each session was videotaped with computer-based video tracking system (EthoVision XT 7.0, Noldus Information Technology Inc, Leesburg, VA, USA) for later analysis by an observer blind to the experimental treatment. The apparatus was wiped with 70% ethanol and air-dried between mice. Recorded moving distance and the time spent in the open arms of the maze was analyzed with Student’s t-test.

### Forced Swimming Test

At 30 days after last closed head injury, mice were subjected to a forced swimming test. Procedures previously reported was used^29^. Briefly, mice were forced to swim for 6 min inside a vertical acrylic cylinder (height, 18 cm; diameter, 11 cm) containing water about 15 cm deep at 25 ± 1 °C. The total duration of immobility time during the 6 min was measured. During the swimming exposure, the mouse was judged to be immobile whenever it remained floating motionless except for movement necessary to keep its head out of the water.

### Immunohistochemistry

After perfusion with PBS followed by 4% paraformaldehyde, brains were processed as either frozen or paraffin-embedded sections. For gross morphological assessment of injury, the paraffin sections were subjected to dewaxing and hydration, and then H&E staining was conducted for 5 min. The slices were differentiated with 0.6% hydrochloric acid alcohol for 30 s, then counterstained in acidified eosin alcohol (pH 4.2) for 3 min, dehydrated and cleared, respectively. To examine astroglosis after repetitive mTBI, the frozen sections were processed for immunohistochemical analysis. A routine staining was performed after a 1-hour blocking step in 2% goat serum. Polyclonal rabbit-anti-GFAP (1:500, Dako, Carpinteria, CA, USA), monoclonal mouse anti-P-Tau at Ser202 (clone CP13, 1:200, a gift from Dr. Peter Davies, Feinstein Institute for Medical Research) were used. The sections were rinsed with phosphate buffered saline (PBS), and then incubated for 2 hr at room temperature with biotin-conjugated secondary antibody and detected by the Vector ABC system (Vector Labs, Burlingame, CA). After three washes in PBS, the sections were mounted with mounting medium. A cohort of four to six mice for each time point and each group was analyzed. For unbiased evaluation of histopathology, images of the slides were scanned using aperio scanner (Leica Microsystems Inc, Buffalo Grove, IL, USA). The computed threshold was determined using Spectrum software and set in order to pick up maximal positive staining of reactive glial fibrillary acidic protein (GFAP) or P-Tau-labeled cells with minimal artifacts. To ensure objective quantification, the same threshold was applied to all brain sections for each region of interest. Positive-stained cells were quantified as a ratio of the number of pixels above the threshold to the total pixels in the selected area.

### Serum T-Tau/P-Tau analysis

As previously described, Enhanced Immunoassay using Multi-Arrayed Fiberoptics (EIMAF) in combination with rolling circle amplification (a-EIMAF) was used to measure the T-Tau and P-Tau levels in mouse serum^1^,^9^. In brief, high-binding 96-well microtiter plates were coated with capture anti-Tau monoclonal antibodies at 6 μg/mL final concentration [Mab DA31 (aa150-190) for T-Tau and Mabs CP13 (pSer-202) for P-Tau]. Following an overnight incubation at 40 ^o^C, unoccupied binding sites were blocked for 1 hr with casein. A 100 μl aliquot of 1:20 diluted serum was added, incubated and followed by the addition of a biotinylated detection Mab DA9 (aa102-140) (100 μl at 4 μg/ml final Mab concentration). Five 10-min washes with PBS containing 0.2% Tween-20 (PBST) were followed with the addition of 100 μl of streptavidin (5 μg/ml) per well and incubation for 1 hr at 37 ^o^C. A biotinylated prostate-specific DNA primer (5’-TTTTTTTGTCCGTGCTAGAAGGAAA-CAGTTAC-3’) (100 μl at 4 μg/ml) was added for 1 hr at 37 ^o^C. Following the addition of a T4-DNA ligase-pretreated prostate DNA template (1 mg/ml), Rolling circle amplification (RCA) was initiated by adding 100 μl of reaction mixture consisting of: φ29 DNA polymerase reaction buffer, bovine serum albumin, nucleotide triphosphates supplemented with dUTP-Texas Red, and φ29 DNA polymerase. Incubation for several hrs is followed by PBST washes, addition of 1N NaOH, neutralization with 1 M Tris-HCl, pH 7.5, heat treatment (100 ^o^C for 15 min) and fluorescence analysis using surround optical fluorescence detection. Each a-EIMAF sample was tested in triplicate and, depending on available sample volumes, duplicated in independent experiments.

### Statistical Analysis

All data are presented as the mean ± standard error of the mean (SEM). Group differences were evaluated by one-way or two-way analysis of variance (ANOVA) or two-tailed Student’s t–test using GraphPad Prism software. Statistical significance was determined by p values of <0.05.

## Additional Information

**How to cite this article**: Yang, Z. *et al.* Temporal MRI characterization, neurobiochemical and neurobehavioral changes in a mouse repetitive concussive head injury model. *Sci. Rep.* **5**, 11178; doi: 10.1038/srep11178 (2015).
