# Corrigendum: Temporal MRI characterization, neurobiochemical and neurobehavioral changes in a mouse repetitive concussive head injury model

**DOI:** 10.1038/srep15922

**Published:** 2015-12-18

**Authors:** Zhihui Yang, Ping Wang, Drake Morgan, Adriaan W. Bruijnzeel, Dan Lin, Jianchun Pan, Fan Lin, Kevin H. Strang, Tyler M. Selig, Pablo D. Perez, Marcelo Febo, Binggong Chang, Richard Rubenstein, Kevin K. W. Wang

Scientific Reports 5: Article number: 11178; 10.1038/srep11178 published online: 06102015; updated: 12182015.

Adriaan W. Bruijnzeel was omitted from the author list in the original version of this Article.

The Author Contributions section now reads:

“Z.Y. and K.W. wrote the main manuscript text. Z.Y. and F.L. prepared Figs 5B, 6 and 7. Z.Y., P.W., D.M., A.B., D.L., J.P., K.S. and T.S. prepared Figs 2, 3 and 4. P.P. and M.F. prepared Fig. 1. B.C. and R.R. prepared Fig. 5A. All authors reviewed the manuscript.”

This has been corrected in the PDF and HTML versions of the Article.

